# OsCIPK9 Interacts with OsSOS3 and Affects Salt-Related Transport to Improve Salt Tolerance

**DOI:** 10.3390/plants12213723

**Published:** 2023-10-30

**Authors:** Zhenling Zhou, Weijie Tang, Zhiguang Sun, Jingfang Li, Bo Yang, Yan Liu, Baoxiang Wang, Dayong Xu, Jianchang Yang, Yunhui Zhang

**Affiliations:** 1 Lianyungang Academy of Agricultural Sciences, Lianyungang 222000, China; zhouzl3716@163.com (Z.Z.); zhiguangsun@126.com (Z.S.); jingfangli@163.com (J.L.); nkykjc19@163.com (B.Y.); ly516.bester@163.com (Y.L.); xudayong3030@163.com (D.X.); 2 Jiangsu Key Laboratory of Crop Genetics and Physiology, Jiangsu Key Laboratory of Crop Cultivation and Physiology, Agricultural College of Yangzhou University, Yangzhou 225009, China; 003335@yzu.edu.cn; 3 Provincial Key Laboratory of Agrobiology, Institute of Germplasm Resources and Biotechnology, Jiangsu Academy of Agricultural Sciences, Nanjing 210014, China; weijiet08@126.com; 4 Zhongshan Biological Breeding Laboratory, No. 50 Zhongling Street, Nanjing 210014, China; 5 Jiangsu Co-Innovation Center for Modern Production Technology of Grain Crops, Yangzhou University, Yangzhou 225009, China

**Keywords:** *OsCIPK9*, *OsSOS3*, sat-related transporters, salt tolerance, rice

## Abstract

Salt is harmful to crop production. Therefore, it is important to understand the mechanism of salt tolerance in rice. *CIPK* genes have various functions, including regulating salt tolerance and other types of stress and nitrogen use efficiency. In rice, OsCIPK24 is known to regulate salt tolerance, but other OsCIPKs could also function in salt tolerance. In this study, we identified another OsCIPK—OsCIPK9—that can regulate salt tolerance. Knockout of *OsCIPK9* in rice could improve salt tolerance. Through expression analyses, *OsCIPK9* was found to be mainly expressed in the roots and less expressed in mature leaves. Meanwhile, *OsCIPK9* had the highest expression 6 h after salt treatment. In addition, we proved the interaction between OsCIPK9 and OsSOS3. The RNA-seq data showed that *OsCIPK9* strongly responded to salt treatment, and the transporters related to salt tolerance may be downstream genes of *OsCIPK9*. Finally, haplotype analyses revealed that Hap6 and Hap8 mainly exist in *indica*, potentially providing a higher salt tolerance. Overall, a negative regulator of salt tolerance, OsCIPK9, which interacted with OsSOS3 similarly to OsCIPK24 and influenced salt-related transporters, was identified, and editing *OsCIPK9* potentially could be helpful for breeding salt-tolerant rice.

## 1. Introduction

An increase in the output of rice, as a staple food feeding almost half the world, will be required over the next 50 years. Therefore, either a higher yield per ha or more cultivated land is needed for a larger harvest. Making full use of the soil would make higher yields achievable. However, salt in saline–alkali soil damages crop production. Salinity includes osmotic stress, ionic toxicity, and nutritional deficiencies, which inhibit rice development [1].

As a major abiotic stressor, researchers have focused on the mechanism of salt tolerance using quantitative trait loci (QTL) mapping [2] and genome-wide association studies (GWASs) [3,4]. With progress in rice genome sequencing, related genes that could be used for rice breeding, such as *SKC1*, have been cloned [5]. *SKC1* is a high-affinity K^+^ transporter which was cloned from Pokkali with extreme salt resistance. OsWRKY53 is a key regulator cloned from GWAS which can regulate the expression of *OsMKK10.2* in promoting ion homeostasis and trans-represses *SKC1* [4]. The superior alleles identified could be useful for breeding rice with salt tolerance. Meanwhile, greater yields would be obtained in saline–alkali soils with the use of salt-tolerant genes [6].

In addition to genes like *SKC1*, *CIPK* family genes have an important role in salt tolerance. CIPK family genes are plant-specific proteins which interact with CBL and serve as major downstream signaling components [7,8]. The CBL-CIPK network plays a vital role in salinity stress, disease defense, drought tolerance, and other stresses [9]. In Arabidopsis, the SOS (Salt Overly Sensitive) pathway was the first well-studied pathway, and this acts as an example for other networks. The AtCBL4–AtCIPK24 complex activates downstream AtSOS1, which affects Na^+^ extrusion and long-distance Na^+^ transport [10]. Also, AtCIPK24 can form an AtCIPK24–GI complex to delay the flowering time under saline conditions [11]. After that, another network, AtCBL10-AtCIPK8, was identified, which also regulates AtSOS1 [12]. In addition to salinity stress, the ATCBL4-AtCIPK6 pathway regulates K^+^ allocation [13]. The AtCBL9-AtCIPK3 network can affect seed dormancy through activating ABR1 in the nucleus [14]. In short, CIPK family genes have multiple functions in Arabidopsis. In rice, CIPKs can phosphorylate many transporters that have multiple functions in various processes, such as nitrogen uptake [15], K^+^ uptake [16], and microbe-associated molecular pattern-induced defense [17]. *OsCIPK9*, *14*, *15* regulates microbe-associated molecular pattern-induced hypersensitive cell death, phytoalexin production, and defense gene expression in cultured cells [18]. Twelve *OsCIPK* genes, including *OsCIPK9*, have been demonstrated to be induced by salinity stress [19]. In addition, *CIPKs* are upregulated during panicle development and abiotic stress [18]. In salt tolerance, *OsSOS2* (*OsCIPK24*) and *OsSOS3* (*OsCBL4*) play vital roles [20]. The calcium-binding protein OsSOS3/CBL4 can sense the cytosolic calcium signal elicited by salt stress, then interact with and activate OsSOS2 (OsCIPK24). Then, activated OsSOS2/OsCIPK24 phosphorylates and activates OsSOS1 to regulate Na^+^ homeostasis and improve rice tolerance to salt stress [21]. In rice, the *CIPK* family has 33 members, with *OsCIPK24* regulating salt tolerance [18], but other *OsCIPKs* which may also have a role in salt tolerance are still unknown. A previous study showed that the mutant *Oscipk9* showed a mild salt tolerance [16], but the possible mechanisms of salt tolerance and the haplotypes of *OsCIPK9* are still unclear.

Here, we proved that OsCIPK9 is a negative regulator of salt tolerance in rice using CRISPR-Cas9. *OsCIPK9* knockouts of rice showed an increased salt tolerance, and overexpression lines were more sensitive. The Na^+^ concentration changed significantly after salt treatment. *OsSOS3* showed a higher expression in the *OsCIPK9-cas* line, and OsSOS3 interacted with OsCIPK9. As determined through RNA-seq data, *OsCIPK9* strongly responded to salt treatment. We found obvious changes in the expression of transporters like *OsKAT1*, especially under saline conditions. Finally, we analyzed the haplotypes of *OsCIPK9* and showed that the haplotype of *OsCIPK9* had an obvious subpopulation classification.

## 2. Results

### 2.1. OsCIPK9 Negatively Regulated Salt Tolerance 

**Figure 1:**
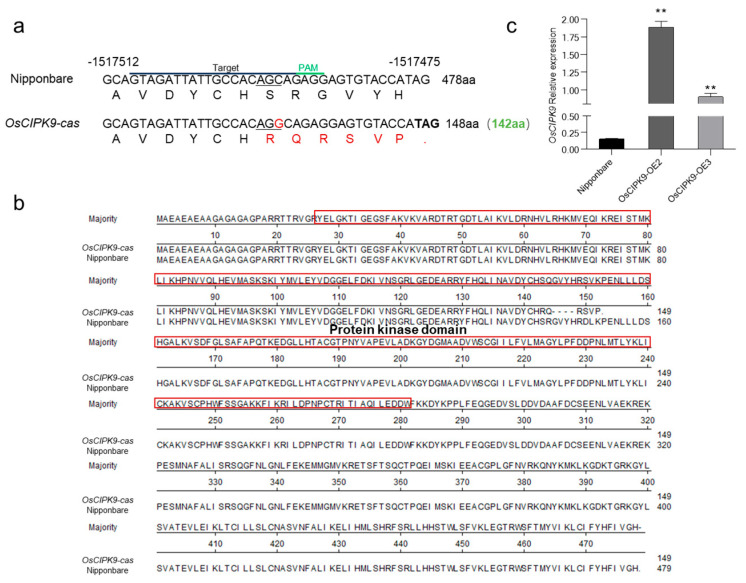
Transgenic line construction. (**a**) Targeted mutagenesis of *OsCIPK9* with CRISPR/Cas9. The mutant alleles have 1 nucleotide insertion in *OsCIPK9-cas*. The base shown in red represents the inserted nucleotide. The amino acids in red represent mutated amino acids. The line below in nucleotides represents the positions which translated the mutated amino acids. (**b**) The result of protein alignment between Nipponbare and *OsCIPK9-cas*. The domain was predicted in Pfam (pfam.xfam.org, accessed on 30 August 2022) presented in red square. (**c**) The expression of Nipponbare and overexpression lines (OsCIPK9-OE2, OsCIPK9-OE3). **: *p* < 0.01. Statistical significance (versus Nipponbare) was calculated using a Student’s *t*-test.

To study the specific role of *OsCIPK9* in rice, we constructed knockout lines using CRISPR-Cas9. One knockout line (*OsCIPK9-cas*) with a base insertion was identified (Figure 1a). The insertion caused a frameshift, and translation stopped after 148 aa (Figure 1a). The protein sequence of the knockout line showed that the domain lacked 140 normal amino acids (143–283) and contained six mutated amino acids (from 143 to 148) compared to that of the wild type (Figure 1b). We also created overexpression lines for further function validation. The overexpression lines OsCIPK9-OE2 and OsCIPK9-OE3 had approximately 9- and 5-fold higher expressions than the wild type (Nipponbare), respectively (Figure 1c).

**Figure 2:**
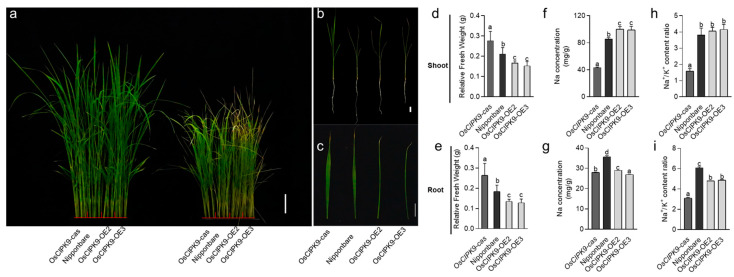
The phenotype of Nipponbare and transgenic lines under 0 and 120 mM NaCl conditions. (**a**–**c**) The phenotype of the wild type (Nipponbare), knockout line (*OsCIPK9-cas*) and overexpression lines (OsCIPK9-OE2, OsCIPK9-OE3) under 0 mM and 120 mM NaCl conditions, bar = 10, 2, 2 cm. (**d**,**f**,**h**) Comparison of Nipponbare, knockout line (*OsCIPK9-cas*) and overexpression lines (OsCIPK9-OE2, OsCIPK9-OE3) in terms of relative fresh weights, Na concentrations per plant and Na^+^/K^+^ content ratios in shoots under 120 mM NaCl conditions. *n* = 3. (**e**,**g**,**i**) Comparison of Nipponbare, the knockout line (*OsCIPK9-cas*) and overexpression lines (OsCIPK9-OE2, OsCIPK9-OE3) in terms of relative fresh weights, Na concentrations per plant and Na^+^/K^+^ content ratios in roots under 120 mM NaCl conditions. *n* = 3. The data are presented as means ± SDs. Statistical significance (versus Nipponbare) was calculated using a Student’s *t*-test. Different letters indicate significant difference, *p* < 0.05.

To verify whether *OsCIPK9* could function in salt tolerance, we treated the transgenic lines and wild type with 0 and 120 mM NaCl. As a result, the knockout line (*OsCIPK9-cas*) showed a higher tolerance to salt treatment, while the overexpression lines were more sensitive (Figure 2a–c). The OsCIPK9-OE2 and OsCIPK9-OE3 lines had a lower relative fresh weight compared with the WT, while that of the knockout line was higher (Figure 2d,e). We also measured the Na^+^ and K^+^ concentrations of whole seedlings. In the shoots, the four lines showed no differences in Na^+^ concentrations and Na^+^/K^+^ content ratios under no-salt conditions (Figure S1), but a discrepancy was observed under salt treatment, in which these values were higher in the OE lines and lower in the knockout lines (Figure 2f,h). In the roots, the Na^+^ concentration of the OE lines was lower than the wild type and the knockout line under no-salt conditions, but displayed almost no difference under saline conditions (Figure S1). The Na^+^/K^+^ content ratio was higher in the OE lines than in the knockout lines (Figure 2g,i). Thus, *OsCIPK9* conferred salt tolerance in rice, and knocking out this gene improved salt tolerance through regulating the Na concentration.

### 2.2. Expression Pattern of OsCIPK9

**Figure 3:**
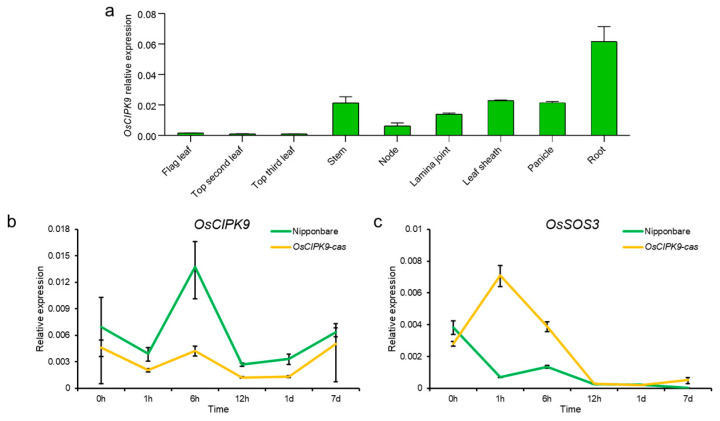
The expression of *OsCIPK9* and *OsSOS3*. (**a**) The expression of *OsCIPK9* in various tissues. (**b**,**c**) Comparison of the expression of *OsCIPK9* and *OsSOS3* between Nipponbare and the knockout line (*OsCIPK9-cas*) under 0 and 120 mM NaCl conditions. *n* = 3. The data are presented as means ± SDs.

To determine the expression pattern of *OsCIPK9*, various tissues were collected and the mRNA abundance of *OsCIPK9* was examined using RT-PCR analysis. The results showed that *OsCIPK9* was most expressed in the roots and least expressed in mature leaves (Figure 3a). Also, we measured the expression at different time points after salt treatment. The results show that *OsCIPK9* had the highest expression 6 h after salt treatment (Figure 3b). The knock-out line showed lower expression from 1 h to 7 days, with the largest difference observed after 6 h. These results indicate that *OsCIPK9* responded to salt treatment, and knocking out the gene reduced the expression under salt treatment.

### 2.3. OsCIPK9 Interacted with OsSOS3

**Figure 4:**
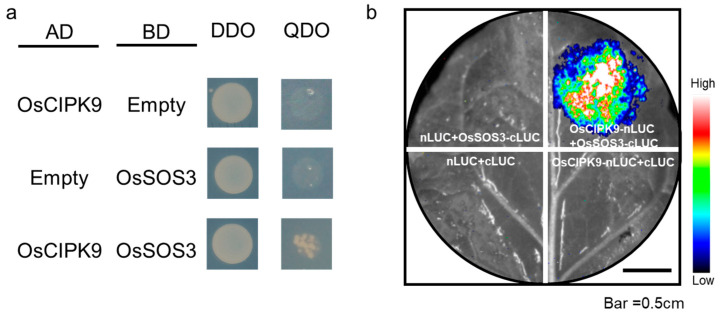
OsCIPK9 physically interacts with OsSOS3. (**a**) Y2H assay showing that OsCIPK9 can interact with OsSOS3 (A). AD, activation domain; BD, binding domain; DDO, SD/–Trp/–Leu; QDO, SD/–Trp/–Leu/–His/–Ade. (**b**) LCI assay showing that OsCIPK9 interacts with OsSOS3 in leaf cells of N. benthamiana. Colored scale bar indicates the luminescence intensity in counts per second (cps). CLUC, C terminus of LUC; NLUC, N terminus of LUC.

According to the change in Na^+^ concentrations in different transgenic lines, including the knock-out and overexpression lines, we examined whether OsSOS3 in the SOS pathway could interact with OsCIPK9 similar to OsSOS2/OsCIPK24. Firstly, *OsSOS3* showed a lower expression in Nipponbare compared with *OsCIPK9-cas*, while *OsCIPK9* showed a higher expression (Figure 3b,c). Thus, we supposed that OsSOS3 may interact with OsCIPK9, playing an opposing role in salt tolerance. To test this hypothesis, we analyzed the interaction between OsSOS3 and OsCIPK9 using a yeast two-hybrid (Y2H) assay. *OsSOS3* strongly interacted with OsCIPK9 (Figure 4a). Also, we verified the interaction between OsSOS3 and OsCIPK9 using an in vivo firefly luciferase complementation imaging (LCI) assay in Nicotiana benthamiana leaf epidermal cells (Figure 4b). Taken together, these results suggest that OsSOS3 could interact with OsCIPK9 in rice.

### 2.4. Transcriptome Analysis of OsCIPK9

**Figure 5:**
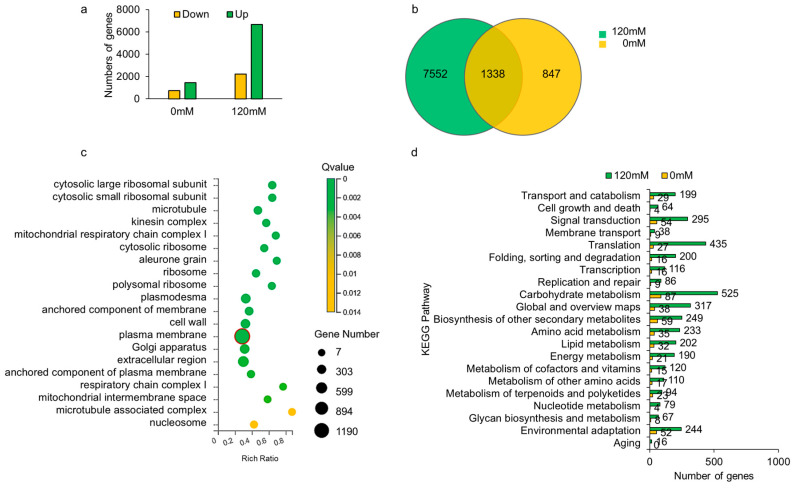
The analysis of RNA-seq data. (**a**) The number of DEGs after 0 mM and 120 mM NaCl treatment. (**b**) The overlap of genes identified after 0 mM and 120 mM NaCl treatment. (**c**) GO analysis of genes identified by RNA-seq. The red circle highlighted the gene number of the plasma membrane identified by GO analysis. (**d**) KEGG analysis of genes identified by RNA-seq.

To identify possible downstream genes influenced by *OsCIPK9*, we performed a transcriptome analysis using knockout lines with different treatments (0 and 120 mM salt). Many upregulated and downregulated DEGs (differential expression genes) were identified, but the number of up- or downregulated genes under NaCl treatment was much greater than under control conditions (Figure 5a). The DEGs included 2285 regulated genes without salt treatment and 9190 genes under salt treatment. Only 1338 genes were found for both treatments (Figure 5b). A GO analysis showed that genes in the plasma membrane of cellular components were identified, but the number of genes under salt treatment (1190) was more than three times that under no salt treatment (358) (Figure 5c and Figure S2). In biological processes, more genes were clustered in cell wall organization under no salt treatment, while the ratio increased under salt treatment. Additionally, more genes were grouped into translation and carbohydrate metabolic processes under salt treatment (Figure S3). The GO molecular function analysis showed that the DNA-binding transcription faction activity process had a large number of genes with a lower Q value under no salt treatment. Under salt treatment, the process with the most regulated genes was structural constituents of the ribosome (Figure S4). Additionally, a KEGG analysis revealed almost no difference between Nipponbare and *OsCIPK9-cas* without NaCl treatment (Figure 5d). A large difference was identified under 120 mM NaCl treatment, as expected (Figure 5d). In the KEGG analysis, almost all processes differed, including transport and catabolism, signal transduction, and membrane transport. The processes consisted of the functions of CIPKs, which have been reported previously [22]. The GO and KEGG results indicated that *OsCIPK9* strongly reacted to salt treatment and played a vital role in salt tolerance, especially in membranes.

**Figure 6:**
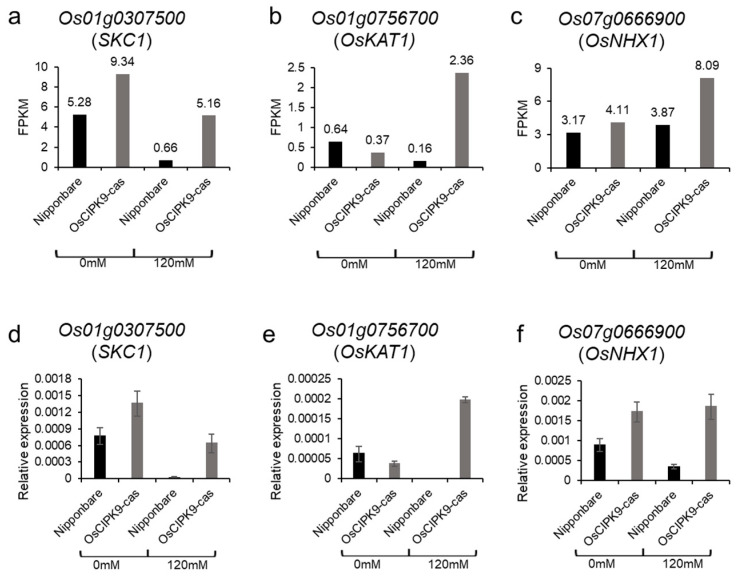
The expression of related transporters. (**a**–**c**) The FPKM of *SKC1*, *OsKAT1*, and *OsNHX1*. (**d**–f) The relative expressions of *SKC1*, *OsKAT1*, and *OsNHX1*.

The RNA-seq analysis showed that *OsCIPK9* affected membrane transport. To identify possible influencing genes in the membrane, we investigated the expression of previously reported transporters, such as *SKC1* and *OsKAT1*, in the RNA-seq data. Luckily, we found that *SKC1*, *OsKAT1*, and *OsNHX1* expression was higher in *OsCIPK9-cas* than in Nipponbare under salt treatment (Figure 6a–c). In particular, the expression of *OsKAT1* rose almost 15-fold under salt treatment, but only 2-fold under no salt treatment (Figure 6b). To verify the expression results, we used real-time PCR to measure the expression of the three transporters and obtained results similar to the RNA-seq data (Figure 6d,e). Therefore, we concluded that *OsCIPK9* conferred salt tolerance by affecting the expression of salt-related transporters expressed in the membrane, like OsKAT1.

### 2.5. The Haplotypes of OsCIPK9

**Figure 7:**
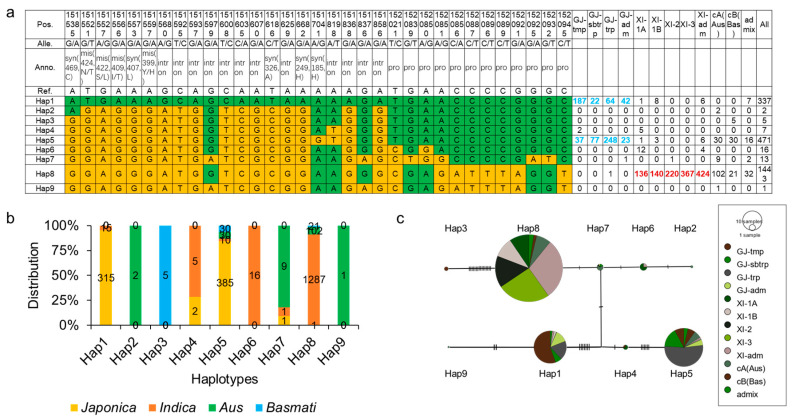
Analysis of haplotypes of *OsCIPK9*. (**a**) Haplotypes of *OsCIPK9* based on the Rice3K database. The green represented the same nucleotides with the reference. The yellow represented the variations compared with the reference. (**b**) The distribution of subpopulations in each haplotype. (**c**) The gene network of different haplotypes. Pos: position; Alle: allele; Anno: annotation; GJ: *japonica*, tmp: temperate, sbtrp: subtropical, trp: tropical, adm: admix. XI: *indica*, Bas: *basmati*, Pro: promoter variation, Syn: synonymous variation, Mis: missense variation, Intron: intron variation.

*OsCIPK9* plays a role in salt tolerance, and the haplotype of *OsCIPK9* may be helpful in breeding or for germplasm improvement in rice. To determine the haplotypes of *OsCIPK9*, we analyzed them using SNP data from the Rice3K database. *OsCIPK9* contains 21 SNPs with 4 missense variants and 12 SNPs in the promoter (Figure 7a). All nine haplotypes were identified based on SNP variants. Hap1 predominantly emerged in *japonica*, mostly in temperate *Japonica* (55.5%), while Hap6 and Hap8 (89.2%) were identified in *indica*. Hap2 and Hap9 were identified only in the *Aus* subpopulation. Hap3 and Hap7 were identified in the *Basmati* subpopulation. Hap5 was identified in various subpopulations but was rarely found in *Indica* (Figure 7b). A previous study demonstrated that the tolerance level of *INDICA* was higher than that of *japonica* at the seedling stage [23]. Therefore, varieties with Hap6 and Hap8 may have a higher salt tolerance than those with Hap1. Subsequently, we analyzed the haplotype network. The results showed that an unknown haplotype connected the haplotypes, mostly in *Japonica* and *Indica* (Figure 7c). Hap7, which was found in almost all *Aus* varieties, was the key haplotype connecting the present haplotypes in *Japonica* and *Indica* (Figure 7c). These results indicate that *OsCIPK9* has a subpopulation classification, and Hap7 is the key haplotype that connects the haplotypes existing in *Japonica* and *Indica*.

## 3. Discussion

In a previous study, *OsCIPK9* was found to regulate ammonium-dependent root growth [15]. Also, AtCIPK23 was found to function in salt tolerance and nitrogen use, including nitrate and ammonium in *Arabidopsis*. Here, knocking out *OsCIPK9* increased the tolerance to salt stress at the seedling stage. Thus, this gene has multiple functions in nitrogen use and salt tolerance. In our results, *OsCIPK9-cas* also showed higher expressions of *OsAMT1.1* and *OsAMT2.1* than Nipponbare under salt stress, especially *OsAMT1.1*, with a two-fold higher expression than the wild type (Figure S5), indicating that *OsCIPK9* plays a role not only in salt tolerance but also in ammonium transport. Overall, *OsCIPK9* could regulate nitrogen use and salt tolerance like *AtCPIK23*.

In this study, OsSOS3 was found to interact with OsCIPK9. In a previous study, OsSOS2 was found to be activated by OsSOS3 [24]. Secondly, *OsKAT1* and some other transporter genes were differentially expressed. In a previous study, researchers found that *CIPK* genes could phosphorylate transporters such as AtCIPK23 and CHL1 in *Arabidopsis* [25]. The phosphorylation of CHL1 has different functions in nitrate transport and sensing. In addition, AtCIPK23 can phosphorylate AtAMT1.1 and influence ammonium uptake [26]. OsSOS2 can phosphorylate OsSOS1, which is a Na^+^/H^+^ antiporter 1, and regulates salt tolerance in rice [27]. Further studies should be performed to reveal whether OsSOS3 can activate OsCIPK9 and whether OsCIPK9 can phosphorylate the transporters detected in our study, which would provide more powerful evidence for salt tolerance.

AtSOS3 (AtCBL4) could function in salt tolerance by interacting with AtSOS2 (AtCIPK24) [10]. In our study, we proved that OsSOS3 could also interact with OsCIPK9. This result indicates that the CBL-CIPK network plays a vitally important role in plant salt tolerance. Also, AtSOS3 (AtCBL4) regulates K^+^ homeostasis through the CBL4-CIPK6-AKT2 pathway [13]. Moreover, AtSOS3 (AtCBL4) was found to be involved in auxin transport [28]. In future studies, more phenotypes may be studied in the OsSOS3-OsCIPK9 pathway.

Other OsCIPKs like *OsCIPK04* also responded to salt treatment according to a previous study [19]. With the development of knockout technology, transgenic lines can be produced more easily than before. Our study showed that *OsCIPK9* could improve salt tolerance using the knockout line. Future studies could focus on other OsCIPKs which may regulate salt tolerance, and rapidly validate the function using CRISP technology. With more OsCIPKs being identified in the future, we may identify a more comprehensive OsCIPK pathway in salt tolerance.

A haplotype analysis showed that lots of variations were present between *japonica* and *indica*. *Japonica* had a different evolutionary process compared to *indica* [29]. Thus, these variations may cause a change in *OsCIPK9*’s function in salt tolerance. Complementation experiments and functional studies of various haplotypes should be conducted in the future. More evidence about the function or causal SNPs of different haplotypes would help in molecular breeding, especially MAS (Marker Assistant Selection).

With progress in gene editing, more editing strategies, such as one-base editing and primer editing, have been used for crop improvements [30]. OsCIPK9 is a negative regulator of salt treatment; therefore, it is better to edit the genomic or promoter region of *OsCIPK9* to produce new alleles for breeding. A haplotype analysis showed that the promoter had more polymorphisms than the gene in our study. Therefore, editing the promoter and creating more lines with differential expressions [31] would be useful for breeding based on the evaluation of other agronomic traits.

## 4. Conclusions

**Figure 8:**
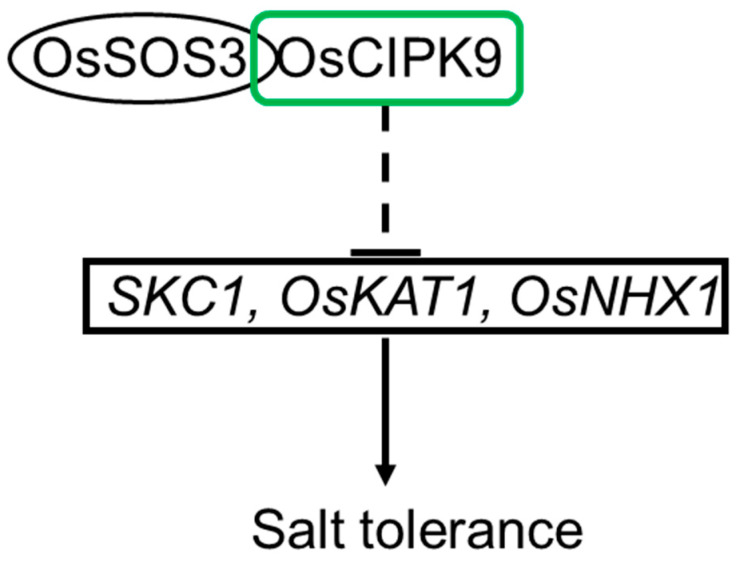
The pathway of OsCIPK9 in the salt tolerance of rice.

In summary, we identified a negative regulator of salt tolerance in rice, OsCIPK9. In addition, we proposed the possible mechanism in which OsCIPK9 is involved through interaction with OsSOS3. The most affected cellular component was that of the plasma membrane, and the downstream genes of OsCIPK9 may be the transporters located in the plasma membrane, like OsKAT1, as observed through RNA-seq analyses. Finally, we suggest the possible pathway of salt tolerance in rice in which OsCIPK9 is involved (Figure 8). Our study shows that other OsCIPKs could also function in salt tolerance, like OsCIPK24. In addition, OsSOS3, which can interact not only with OsCIPK24 but also with OsCIPK9, regulates salt tolerance in rice. More importantly, our study investigated more OsCIPKs which may regulate salt tolerance in rice.

## 5. Materials and Methods

### 5.1. Plant Materials and Transgenic Lines Construction

The Line *japonica* Nipponbare was used as the wild type, and CRISPR/Cas9 technology was used to produce the knockout line (*OsCIPK9-cas*). The pam sequence is shown in Figure 1a. The full CDS of *OsCIPK9* was cloned from the cDNA of Nipponbare, and the pCUbi1390 recombination vector was constructed. Overexpression lines were developed using *Agrobacterium*-mediated genetic transformation. Two independent overexpression lines (OsCIPK9-OE2 and OsCIPK9-OE3) were isolated from transgenic lines. The expression of overexpression lines was determined using real-time PCR.

### 5.2. Salt Treatment and Phenotypic Identification

The seeds of all lines were geminated and grown in Yoshida nutrition solution for 14 days. NaCl concentrations of 120 mM and 0 mM (CK) were used for the treatment and control, respectively. The traits, including fresh weight and Na^+^ and K^+^ concentrations, were measured 7 days after NaCl treatment. Relative fresh weight = fresh weight under no salt conditions − fresh weight under salt treatment. Na^+^ and K^+^ concentrations were determined according to the method described by Wang et al. [32].

### 5.3. Y2H Assays

A Y2H assay was performed with a MatchMaker GAL4 Two-Hybrid System (Clontech, Mountain View, CA, USA, https://www.takarabio.com/, accessed on 14 August 2023). The full-length coding sequences of genes (*OsCIPK9* and *OsSOS3*) of interest were cloned from the cDNA of Nipponbare into pGADT7 and pGBKT7 (Clontech, Mountain View, CA, USA, https://www.takarabio.com/, accessed on 14 August 2023), and different combinations of constructs were transformed together into the yeast (*Saccharomyces cerevisiae*) AH109 strain. Positive transformants were selected on synthetic dropout (SD/−Leu/−Trp, DDO) nutrient media, while the interactions were screened in SD medium (SD/−Leu/−Trp/−His/−Ade, QDO).

### 5.4. Firefly LCI Assays

The coding sequences of *OsCIPK9* and *OsSOS3* were cloned into the pCAMBIA-nLUC or pCAMBIA-cLUC vectors. These constructed vectors were introduced into the Agrobacterium tumefaciens strain EHA105. Various combinations of EHA105 strains were used to infiltrate *N. benthamiana* leaves. The relative LUC activity was measured by using a Nightshade LB 985 system (Berthold Technologies, Baden, Germany, 10 August 2023), as described previously [33].

### 5.5. RNA-Seq

Additionally, the roots of different lines under 0 and 120 mM NaCl treatments were collected for RNA-seq analysis. The total RNA of three independent plants was isolated using a plant RNA purification reagent (Invitrogen, Shanghai, China, 10 November 2022). RNA-seq was performed using the BGI T7 platform, and analyses of DEGs (Differentially Expressed Genes), KEGG (Kyoto Encyclopedia of Genes and Genomes), and GO (Gene Ontology) were performed using Dr. Tom website produced by BGI, China (https://biosys.bgi.com, accessed on 20 January 2023). The expression of all genes produced via RNA-seq is listed in Table S1.

### 5.6. Real-Time PCR

The total RNA of different tissues and roots at different time points was extracted using an RNA prep Pure Plant Kit (Tiangen Biotech, Beijing, China). Then, ~1 μg of total RNA was reverse-transcribed into cDNA using a PrimeScipt^TM^ Reverse Transcriptase kit (Takara, Shiga, Japan, www.takarabio.com, accessed on 5 March 2023). Quantitative RT-PCR assays were performed using an SYBR Premix Ex Taq™ kit (Takara, Shiga, Japan, www.takarabio.com, accessed on 5 March 2023). Real-time PCR was performed in a real-time PCR machine (I-Cycle, Bio-Rad, Hercules, CA, USA ). The primers are listed in Table S2. The rice *UBQ* (*Os03g0234350*) gene was used as an internal control.

### 5.7. Haplotype Analysis

SNP data of Rice3K were downloaded from the Rice SNP-Seek Database (https://snp-seek.irri.org/, accessed on 5 June 2022). The haplotypes were separated based on all SNP variants of the *OsCIPK9* gene and promoter (~2K upstream sequence of *OsCIPK9*). The subpopulations of rice were divided into *japonica* (temperate, subtropical, tropical, and admix), *indica* (1A,1B,2,3, and admix), Aus, Basmati, and admix. The haplotype network was constructed using PopART software (https://popart.maths.otago.ac.nz/, accessed on 5 July 2022) [34].

## Data Availability

The datasets supporting the conclusions of this article are included within the article and Supplementary Materials.

## Supplementary Materials

The following supporting information can be downloaded at: https://www.mdpi.com/article/10.3390/plants12213723/s1, Figure S1: Comparison of Nipponbare (Nip), knock-out line (*OsCIPK9-cas*) and overexpression lines (OsCIPK9-OE2, OsCIPK9-OE3) Na concentrations per plant and Na^+^/K^+^ content ratios in shoot and root under 0 mM NaCl conditions; Figure S2: The result of GO cellular component analysis between *OsCIPK9-cas* and Nipponbare under 0 mM NaCl treatment; Figure S3: The result of GO biological process analysis between *OsCIPK9-cas* and Nipponbare under 0 mM and 120 mM NaCl treatment; Figure S4: The result of GO molecular function analysis between *OsCIPK9-cas* and Nipponbare under 0 mM and 120 mM NaCl treatment; Figure S5: The FPKM of *OsAMT1.1* and *OsAMT2.1* in Nipponbare and knock-out line (*OsCIPK9-cas*) under 0 mM and 120 mM NaCl conditions; Table S1: The expression of all genes produced by RNA-seq; Table S2: The list of primers.
