# Supplementary material for: OsCIPK9 Interacts with OsSOS3 and Affects Salt-Related Transport to Improve Salt Tolerance
Source: Plants (Basel). 2023 Oct 30;12(21):3723. doi: 10.3390/plants12213723 (PMC10647249; doi:10.3390/plants12213723)
Supplement: Supplementary file 1 [file plants-12-03723-s001.zip › Supplementary data.pdf]

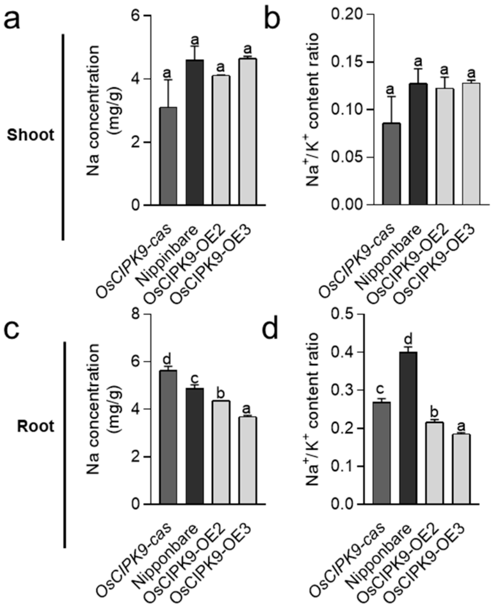

Figure S1. Comparison of Nipponbare (Nip), knock-out line (*OsCIPK9-cas*) and overexpression lines (OsCIPK9-OE2, OsCIPK9-OE3) Na concentrations per plant and Na^{+}/K^{+} content ratios in shoot and roo under 0mM NaCl conditions. a-b: The comparison in shoot. c-d: The comparison in root. n = 3. Data are presented as means ± SD. Statistical significance (versus the Nip) were calculated with Student's *t*-test. Different letters indicate significant difference, $P < 0.05$.

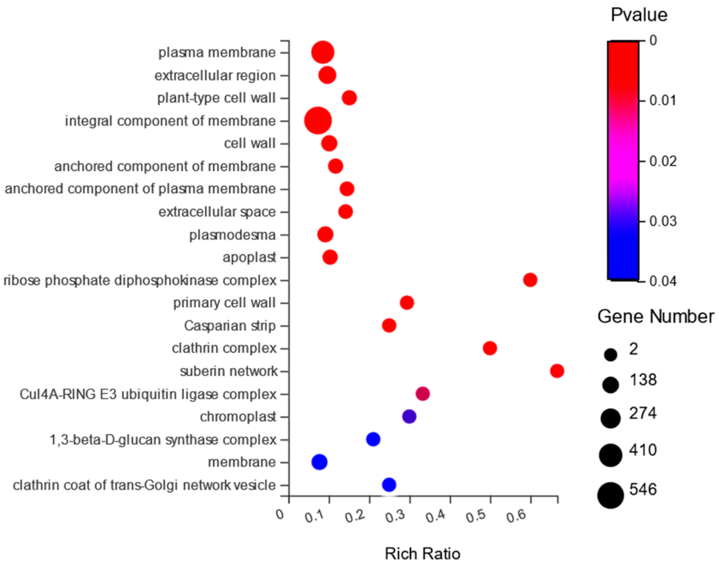

Figure S2. The result of GO cellular component analysis between *OsCIPK9-cas* and Nipponbare under 0mM NaCl treatment.

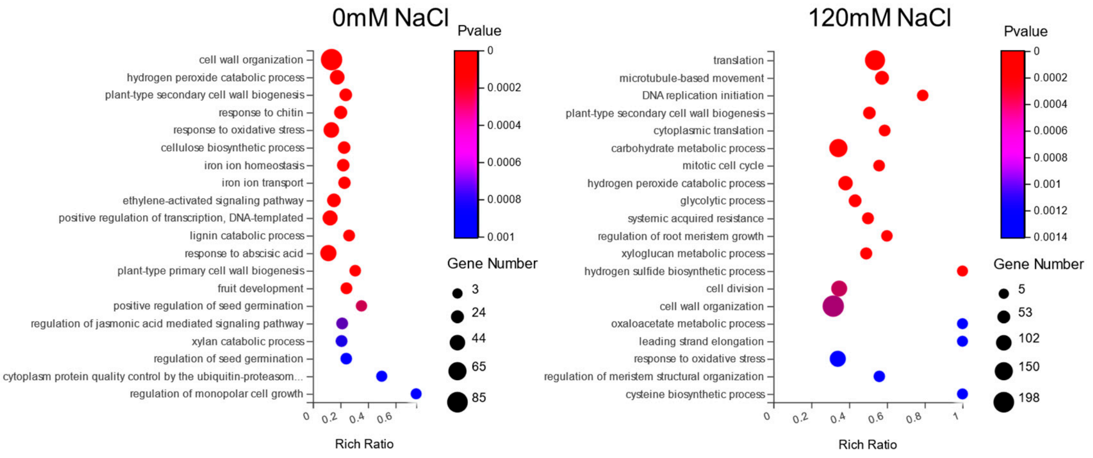

Figure S3. The result of GO biological process analysis between *OsCIPK9-cas* and Nipponbare under 0mM and 120mM NaCl treatment.

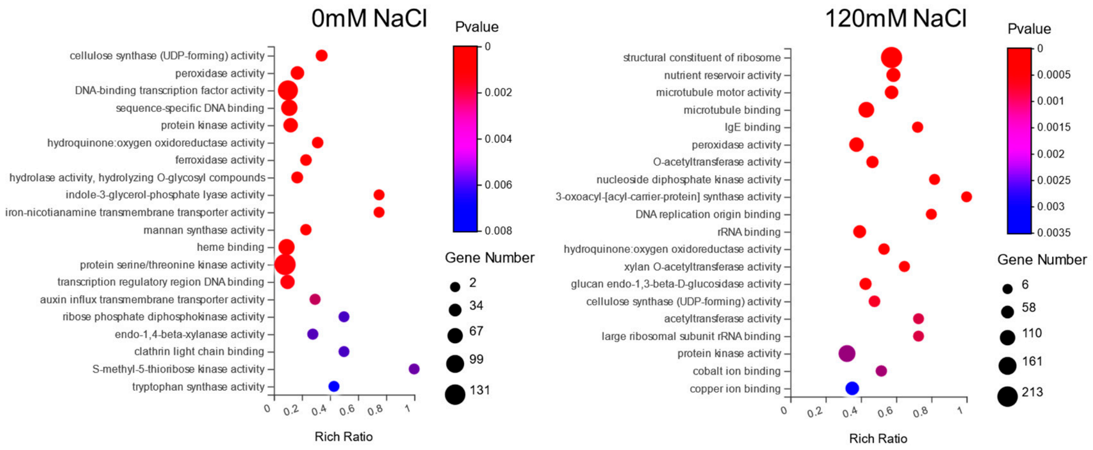

Figure S4. The result of GO molecular function analysis between *OsCIPK9-cas* and Nipponbare under 0mM and 120mM NaCl treatment.

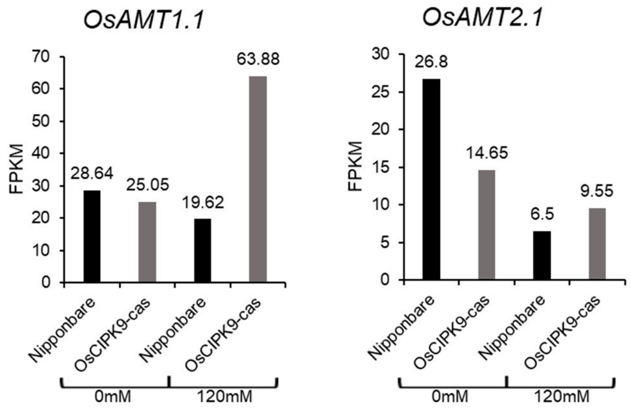

Figure S5. The FPKM of *OsAMT1.1* and *OsAMT2.1* in Nipponbare and knock-out line (*OsCIPK9-cas*) under 0mM and 120mM NaCl conditions.
